# Advances in Hydrogel-Integrated SERS Platforms: Innovations, Applications, Challenges, and Future Prospects in Food Safety Detection

**DOI:** 10.3390/bios15060363

**Published:** 2025-06-05

**Authors:** Xorlali Nunekpeku, Huanhuan Li, Ayesha Zahid, Chenhui Li, Wei Zhang

**Affiliations:** School of Food and Biological Engineering, Jiangsu University, Zhenjiang 212013, China

**Keywords:** hydrogel, surface-enhanced Raman scattering (SERS), food safety detection, nanoparticle stabilization, smart materials

## Abstract

*Background*: Food safety remains a global concern due to biological and chemical contaminants, including adulterants, pathogens, antibiotic residues, and pesticides. Traditional detection methods are accurate but limited by time requirements, complex sample preparation, high costs, and poor field applicability. Surface-Enhanced Raman Spectroscopy (SERS) offers non-destructive analysis with low detection limits and high specificity, yet conventional SERS substrates face challenges with reproducibility, nanoparticle aggregation, and sensitivity in food matrices. Hydrogels have emerged as supporting materials for SERS due to their water content, tunable porosity, flexibility, and ability to entrap plasmonic nanostructures. *Scope and Approach*: This review examines recent advances in hydrogel-integrated SERS platforms for food safety applications. The three-dimensional structure of hydrogels enables homogeneous distribution of metal nanoparticles, prevents aggregation, and offers analyte enrichment. We analyze material design, functionalization strategies, and how hydrogel properties—crosslinking density, porosity, surface charge, and nanoparticle distribution—influence SERS performance in food matrices. *Key Findings and Conclusions*: Hydrogel-integrated SERS platforms demonstrate superior performance in detecting various food contaminants—including pesticides, adulterants, and additives—in real food matrices, often achieving detection limits in the nanomolar to picomolar range, depending on the analyte and substrate design. Current limitations include storage stability concerns, batch-to-batch variability, and regulatory acceptance hurdles. Future research directions should focus on multiplex detection capabilities, integration with smart sensing technologies, and industrial scalability to facilitate practical deployment in global food safety monitoring across diverse supply chains.

## 1. Introduction

Food safety represents one of the most significant global public health challenges of our time, with foodborne illnesses affecting approximately 600 million people annually according to the World Health Organization (WHO) [1,2,3]. What was once primarily a concern about microbial contamination has evolved into a complex landscape encompassing both biological threats (Salmonella, E. coli, Listeria, and viruses) and chemical contaminants (pesticides, heavy metals, and adulterants) that may be introduced at any point from farm to table [4,5,6,7]. As food supply chains become increasingly globalized and consumer demand for safety assurance grows, the limitations of conventional detection methods have become increasingly apparent [8,9].

Traditional food safety monitoring approaches—such as microbial culturing, enzyme-linked immunosorbent assays (ELISA), and polymerase chain reaction (PCR)—while foundational to current detection systems, suffer from critical drawbacks that limit their practical application. These methods typically require 24–72 h for results, depend on specialized laboratory infrastructure, involve destructive sample preparation, and remain poorly suited for the real-time, field-based monitoring essential in today’s rapid food distribution networks. The economic and public health consequences of these limitations are substantial, with delayed detection leading to widespread outbreaks, costly recalls, and diminished consumer confidence [10,11,12].

Surface-Enhanced Raman Spectroscopy (SERS) has emerged as a transformative solution that directly addresses these challenges. Unlike conventional methods, SERS can provide molecular fingerprinting of contaminants at trace concentrations with minimal sample preparation, offering detection sensitivity orders of magnitude greater than standard spectroscopic techniques through localized surface plasmon resonance in metal nanostructures [13,14,15]. This capability is particularly valuable for food safety applications, where rapid identification of contaminants can prevent widespread distribution of adulterated or unsafe products [16,17,18]. However, despite its tremendous potential, widespread adoption of SERS has been hindered by persistent challenges—particularly substrate instability, signal variability, and nanoparticle aggregation in the complex chemical environments typical of food matrices [19,20,21].

The integration of hydrogels into SERS platforms represents a paradigm shift in addressing these fundamental limitations. Hydrogels—three-dimensional polymeric networks with high water content—provide an ideal microenvironment for stabilizing plasmonic nanoparticles while maintaining optimal interparticle distances for electromagnetic enhancement. Their unique structure also enables selective filtration of target analytes, excluding larger interfering molecules common in food matrices. This selective permeability, combined with the ability to incorporate functional recognition elements, creates sensing platforms specifically tailored to food contaminant detection that outperform conventional SERS substrates in both sensitivity and reliability [22,23,24]. Natural polymers such as chitosan, alginate, and cellulose, along with synthetic and hybrid hydrogels, have been extensively explored for these applications, offering distinct advantages in biocompatibility, biodegradability, and functionalization capabilities [25,26,27]. The incorporation of recognition elements—such as aptamers, antibodies, or molecularly imprinted polymers—further enhances selectivity, enabling the detection of specific contaminants even in heterogeneous food samples. Most importantly, hydrogel-SERS platforms bridge the gap between laboratory capabilities and practical field deployment, offering stable, user-friendly systems suitable for on-site monitoring throughout the food supply chain [28,29,30,31].

Despite these advances, several critical challenges remain. While proof-of-concept studies have demonstrated the potential of hydrogel-based SERS platforms, issues such as reproducibility, long-term stability under varying environmental conditions, and the scalability of fabrication methods need to be addressed [32,33]. Additionally, integration with portable, user-friendly detection systems is crucial for on-site, rapid food safety testing, particularly in the context of global food supply chains [34,35]. Furthermore, the selectivity of these systems in complex food matrices remains a key issue, as traditional size-exclusion and charge-based interactions often struggle to distinguish between closely related contaminants in multi-contaminant scenarios [36,37].

**Figure 1:**
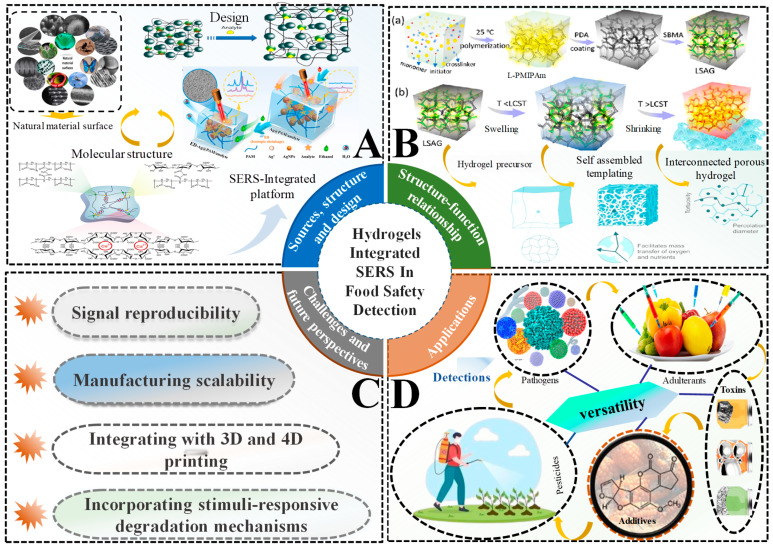
Overview of hydrogel-integrated SERS (Surface-Enhanced Raman Scattering) systems for food safety detection. (**A**) *Sources, structure, and design*: includes natural material surfaces, molecular structures, and schematic of a SERS-integrated hydrogel platform for analyte detection; (**B**) *Structure–function relationship*: (**a**) Synthesis of L-PNIPAm hydrogel via polymerization at 25 °C, followed by polydopamine (PDA) coating and sulfobetaine methacrylate (SBMA) functionalize to form LSAG hydrogel. (**b**) Thermoresponsive behavior of LSAG hydrogel: swelling at temperatures below the lower critical solution temperature (T < LCST) and shrinking at temperatures above (T > LCST); interconnected porous structure facilitates mass transfer; (**C**) *Challenges and future perspectives*: includes signal reproducibility, scalability of manufacturing, integration with 3D/4D printing, and development of stimuli-responsive degradation mechanisms; (**D**) *Applications*: shows the versatility of hydrogel-SERS platforms for detecting a wide range of food contaminants, including pathogens, pesticides, toxins, adulterants, and additives.

This review offers a comprehensive analysis of hydrogel-integrated SERS platforms in food safety detection, focusing on the following: (1) the critical material characteristics—such as crosslinking density, porosity, surface charge, hydrophilicity, hydrophobicity, and nanoparticle distribution—that optimize SERS performance; (2) the fundamental interactions between hydrogels and plasmonic nanoparticles that enhance sensitivity, stability, and specificity; (3) practical applications in detecting a wide range of contaminants, including pesticides, toxins, adulterants, and illegal additives across diverse food matrices; (4) the key challenges related to reproducibility, scalability, nanoparticle stability, molecular selectivity, and regulatory compliance; and (5) future prospects driven by emerging technologies, including artificial intelligence, Internet of Things integration, smart materials, and advanced manufacturing techniques to overcome current limitations. The insights presented aim to support researchers, industry practitioners, and regulatory bodies in advancing reliable, real-time food safety monitoring solutions. A schematic summary of the sources, design strategies, fabrication techniques, applications, and challenges of hydrogel-integrated SERS platforms is illustrated in Figure 1.

## 2. Hydrogels: Concept and Design

### 2.1. The Role of Hydrogels in SERS Technology

Hydrogels bridge soft material science and applied design, offering a flexible platform for sensor applications, particularly in food safety. Most rigid materials have limited practical applications due to their fixed structure; in contrast, hydrogels offer tunable properties that include flexibility, high-water content, and porosity that make them well-suited to biological environments and environments engineered for signal sensitization, such as pH-, temperature-, or analyte-responsive conditions that enhance SERS performance [38,39].

Originally simple water-holding polymers, hydrogels have evolved into complex networks with multi-functional capabilities that enhance detection sensitivity and selectivity [40,41]. Their hydrated porous structure creates poroelastic and viscoelastic properties similar to biological tissues, making them excellent substrates for molecular interactions [42]. Hydrogels provide controlled permeability, allowing for selective filtration, with a stimuli responsiveness that endures the fluctuating environments while measuring food exposures [43,44].

Recent innovations incorporate nanoscale elements—nanopores, nanofibers, and plasmonic nanoparticles—within polymer networks [45,46]. These nanoscale hydrogels can provide much higher surface area (>500 m^2^/g versus <10 m^2^/g for conventional hydrogels), which improves functional performance [47]. High surface-to-volume ratios improve interactions with trace contaminants, increase detection kinetics, and control interactions with food components in hydrogels. In SERS, hydrogels with plasmonic nanoparticles produce electromagnetic field hotspots that enhance the Raman signal [48], while the matrix prevents nanoparticle aggregation and maintains signal reproducibility [49,50].

Hydrogels can also respond to food-related external stimuli, undergoing transformations in response to pH changes in fermented foods, temperature variations during processing, ionic strength in preserved products, or bacterial metabolites [51]. This responsiveness enables smart sensing platforms that adapt to sample conditions and visually indicate contamination without complex instrumentation [52].

Hydrogel sensing technology has evolved through three phases: initial structural designs mimicking natural structures [53], followed by functional elements enabling stimuli responsiveness and improved selectivity [54], and most recently, integrative designs combining multiple functional properties like self-healing and selective analyte transport. These advancements enhance time-sensitive and consistent food safety monitoring in complex matrices [55].

### 2.2. Hydrogel-Based Materials for SERS Applications

Hydrogels are critical to improving food safety detection using SERS, and their various origins dictate the high functional diversity of their material nature. Materials are divided into three categories: natural, synthetic, and hybrid systems. Each of these has various positive effects, enhancing sensing platform performance and flexibility by enabling better contaminant identification (Table 1).

#### 2.2.1. Natural Materials

**Figure 2:**
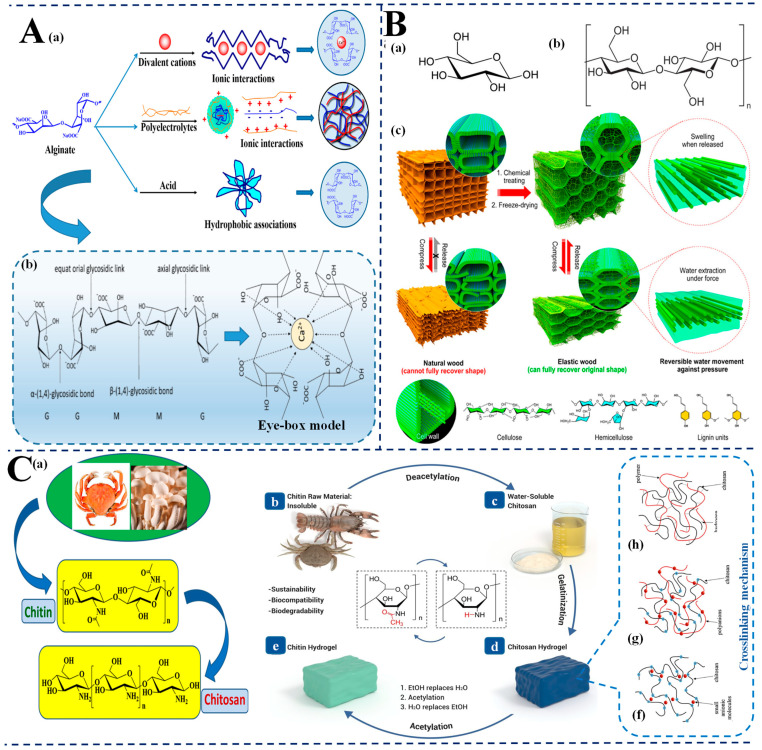
Natural-material-based hydrogels for SERS food safety applications. (**A**) Alginate hydrogels: (**a**) Gelation mechanisms of alginate via ionic interactions with divalent cations, polyelectrolytes, and acids, forming tunable 3D networks; (**b**) Chemical structure of alginate and formation of “egg-box” model through Ca²⁺ crosslinking of α-L-guluronic acid (G) blocks; (**B**) Cellulose-based hydrogels: (**a**,**b**) Molecular structures of cellulose and its polysaccharide chains; (**c**) Schematic comparison of natural and treated elastic wood: structural recovery and water mobility due to cellulose network integrity, enabling reversible shape change and water responsiveness; (**C**) Chitosan hydrogels: (**a**) Sources of chitin from crustaceans and fungi; (**b**,**c**) Conversion of chitin (water-insoluble) to chitosan (water-soluble) via deacetylation; (**d**,**e**) Hydrogel formation via acetylation and sol–gel transformation; (**f**–**h**) Crosslinking strategies and intermolecular interactions enabling chitosan’s gel structure and analyte-binding capabilities.; adapted from [71,72,73,74,75,76,77].

Natural-material-based hydrogels, such as alginate, cellulose, and chitosan, are extensively used in SERS-based food contaminant analysis due to their biocompatibility, selective permeability, and inherent molecular recognition properties. Alginate, in particular, is valued for its tunable physical properties and multifunctional crosslinking characteristics (Figure 2A). It has a backbone made of β-D-mannuronic acid (M) and α-L-guluronic acid (G) residues whose arrangement determines gelation behavior and mechanical strength [56]. The inclusion of G-blocks facilitates ionic crosslinking with Ca^2^⁺ ions via the classical “egg-box” model, forming porous 3D networks that are permeable to the diffusion of analytes. This tunability allows for modifiable stiffness (0.2–350 kPa), swelling (20–300 g/g), and pore size (5–200 nm) and hence renders alginate highly appropriate for the detection of hydrophilic pollutants in aqueous food matrices [57]. Also, its carboxyl-rich nature favors chemical modification, favoring selective binding of the analyte and enhanced SERS enhancement [58].

Cellulose, the renewable natural biopolymer with the highest abundance, enjoys exceptional mechanical toughness and stability—attributes essential for the formation of durable SERS-active substrates (Figure 2B). Its β-1,4-linked D-glucopyranose architecture creates fibrillar assemblies ranging from nano- to macro-scale, enhancing dimensional stability and hydrogen-bonded networks [47,59]. Nanocellulose yields hydrogels with large surface areas (200–700 m^2^/g) and retains reactive hydroxyl groups for nanoparticles immobilization or further functionalization [60,61]. Bacterial cellulose, produced by Gluconacetobacter xylinus, offers better crystallinity (>60%) and water-holding capacity (>99%), optimal for flexible, high-performance substrates of SERS for direct-contact food sensing [62,63].

Chitosan, which is generated through chitin deacetylation, is particularly valuable in SERS food safety systems due to its cationic nature under weakly acidic conditions (Figure 2C). It is made up of D-glucosamine and N-acetyl-D-glucosamine that are linked via β-1,4 glycosidic linkages [64,65,66]. In weakly acidic environments (pH < 6), protonated amino groups impart a polycationic nature, which facilitates strong electrostatic interaction with anionic species like foodborne pathogens and bacterial cell walls [67,68]. Ionic, metal-ion, or covalent crosslinked chitosan hydrogels confer inherent antimicrobial activity—disrupting microbial membranes—thus applicable in the inhibition and detection of food safety [69].

These biopolymer hydrogels, such as alginate, cellulose, and chitosan, offer a promising platform for SERS-based food contaminant analysis [70]. Their abundant functional groups, biodegradability, and tunable structural properties make them suitable for sensitive, selective, and sustainable monitoring methods. Further exploration of underutilized biopolymers can continue to diversify their application to useful food safety diagnosis.

#### 2.2.2. Synthetic Materials

Synthetic hydrogels are key contributors to SERS-based food safety detection owing to their tunable architecture, stable chemical structure under external forces, and superior mechanical properties [78]. They are designed to complement the limitations of natural hydrogels, whether due to variability, weak mechanical properties, or limited functionalisation. Synthetic hydrogels can retain a reliable performance across the varied analytical conditions that may exist. Synthetic hydrogels possess tunable porosity, crosslink density, and chemical modifications which allow for fine-tuning the analyte diffusion, stabilization of nanoparticles, and hotspots for obtaining sensitive and reproducible SERS signals [79,80]. Of the more commonly used synthetic hydrogels, polyvinyl alcohol (PVA) has a strong influence due to its relatively high water retention capacity (70% to 95%) and freeze–thaw crosslinking capabilities which create stable porous networked structure for achieving uniform distribution of plasmonic nanoparticles and analyte transport, and improved reproducibility of SERS signals in food matrices [81,82]. The degree of hydrolysis of PVA influences its crystallinity, solubility, and mechanical properties [83]. PVA salts with a higher crystallinity have greater mechanical stability, elastic moduli in the order of 0.1 to 1 MPa, subsequently depending on the crosslinking conditions and degree of polymer concentration in hydrogel structures [84]. In food safety applications where SERS is applied, PVA hydrogels would typically be produced by means of physical crosslinking, notably freeze–thaw cycling. This allowed for stabilization of a porous framework with pore sizes between 1 and 10 μm, which increases analyte access, curtails nanoparticle aggregation, and ensures SERS signal reproducibility and sensitivity within complex food matrices [85]. Furthermore, the presence of hydroxyl groups in PVA lends itself readily to chemical modification, providing the opportunity to further functionalize the hydrogel with molecular recognition elements, which improves the specificity of target analytes [86]. Polyethylene glycol (PEG) is also a common synthetic polymer that possesses good antifouling properties and tunable mesh sizes that would reduce background noise while evaluating complex protein-rich samples, and can allow specific molecular recognition through copolymerization if required [87,88]. Graphene oxide (GO)-reinforced hydrogels broaden the use of synthetic platforms, given their enhancement of mechanical strength, thermal degradation stability, and SERS enhancement, attributed to GO’s large surface area, with a broad range of surface functionality (~2630 m^2^/g) [89,90]. Molecularly imprinted polymer (MIP) hydrogels further elicit selective recognition properties, given template-specific binding sites, allowing for mimicry of antibody-like specificity towards undesired contaminants such as pesticides and adulterants [91,92,93,94,95]. Overall, the versatility, reproducibility, and functional adaptability of synthetic hydrogels make them indispensable in the development of high-performance SERS platforms for food contaminant monitoring.

**Table 1:** Common hydrogel materials for SERS substrates, their properties, advantages, and limitations in food contaminant detection.

Material Category	Specific Material	Source/ Composition	Key Properties	Material Types	Advantages	Limitations	References
Natural Materials	Chitosan	Crustacean shells	Cationic, antimicrobial; biodegradable	Biopolymers	Selective binding; inherent antimicrobial activity	Limited stability at low pH; batch variability	[68,96]
	Cicada wings	Cicada exoskeleton	Hierarchical nanostructure; light trapping	Natural photonic template	Enhanced light absorption; ordered nanostructure	Fragility; limited scalability	[97,98,99]
	Alginate	Brown seaweed	Anionic; ionic cross-linkable	Polysaccharides	Facile gelation; biocompatible; thickening stability	Poor mechanical strength; limited shelf-life	[100,101]
	Mussel shell	Marine mollusk shell	Layered nacre structure; high mechanical strength	Inorganic template	Structural ordering; high mechanical durability	Brittle; complex processing	[102,103]
	Cotton swabs	Cotton plant fibers	High porosity; hydrophilicity; cellulose-based	Cellulose-based template	Low cost; easy availability; high surface area	Poor structural stability; batch variability	[104,105]
	Gelatin gel	Animal-derived collagen	Biodegradable; thermoresponsive; forms physical hydrogels	Biopolymers	Good film-forming ability; edible; biocompatible	Temperature sensitive; poor mechanical strength	[106,107,108]
	Diatom frustule	Diatom microalgae	Hierarchical porous silica structure; optical properties	Silica-based template	High surface area; light manipulation capability	Fragility; batch variability	[109]
	*Canna generalis* leaf	Plant leaf microstructure	Micro/nano ridges; super hydrophobicity	Natural template	Antireflective; self-cleaning surface	Limited mechanical robustness	[110]
	Fish scale substrate	Collagen-hydroxyapatite composite	Hierarchical structure; transparency; biocompatible	Natural composite	Renewable source; high flexibility; biocompatibility	Processing complexity; low mechanical strength	[111,112]
	Bacterial nanocellulose	Bacterial fermentation	Highly crystalline; fibrous network	Nanocellulose	High mechanical strength; thermal stability	Slow production; high cost	[113,114]
	DNA-based	Synthetic or extracted	Sequence-specific recognition	Nucleic acids	High selectivity; programmable	Temperature sensitive; enzyme degradation	[9,115,116,117]
	Silk Fibroin	Silkworm cocoons	β-sheet structures	Proteins	Stability across pH range; optical clarity	Complex extraction; high cost	[118,119]
	Cellulose (Microcrystalline cellulose)	Plant fibers	Biodegradable, high mechanical strength	Polysaccharides	Biodegradable; high strength	Limited availability; expensive processing	[120,121,122,123]
Synthetic Materials	PVA	Polyvinyl alcohol	High transparency; tunable crosslinking	Synthetic polymers	Optical clarity; good mechanical properties	Limited selectivity	[124,125]
	PEG	Polyethylene glycol	Hydrophilic; low fouling	Polymers	Reduced non-specific binding	Limited functionality	[126,127]
	GO-based	Graphene oxide	High surface area; conductive	Nanomaterials	Enhanced EM field; improved signal	Complex synthesis; batch variability	[128]
	Pluronic-based	PEO-PPO-PEO copolymers	Thermoresponsive; amphiphilic	Block copolymers	Dynamic structure; capture-release	Limited temperature range	[129]
	MIP	Templated polymers	Highly selective binding sites	Synthetic polymers	Antibody-like specificity; stability	Template removal challenges	[95,130,131]
	Conductive Polymers	PANI, PPy, PTh	Electrically conductive; redox active	Conductive polymers	Dual optical-electrical detection	Environmental sensitivity	[132,133]
	Zwitterion	pCBMA, pSBMA	Ultra-low fouling	Polymers	Reduced non-specific binding	Complex synthesis; higher cost	[134]
Hybrid Systems	Natural-Synthetic Composites	Chitosan, polyvinyl alcohol (PVA), alginate	Enhanced mechanical properties	Polysaccharides; Synthetic Polymers	Structural reinforcement with bio recognition	Optimizing component ratios	[135]
	IPNs (Interpenetrating Polymer Networks)	Gelatin-polyacrylamide	Interlaced networks	Hydrogels	Improved mechanical strength	Complex fabrication	[136,137]
	Graphene Oxide-Polymer Hybrid Hydrogels	Graphene oxide, polymers (e.g., PVA)	High mechanical strength; conductivity; bioactivity	Nanomaterials; synthetic polymers	High mechanical strength; flexibility	Processing complexity; limited scalability	[138]
	Ti_3_C_2_/Sodium Alginate Hybrid Hydrogel	Ti_3_C_2_ MXene, Sodium Alginate	Highly conductive; biocompatible, electrochemical sensing	2D materials; natural polymer	High conductivity; excellent biocompatibility	Fabrication complexity; material degradation	[139]
	Serotonin-Modified Gelatin and Oxidized Hyaluronic Acid Hydrogel	Serotonin-modified Gelatin, Oxidized Hyaluronic Acid	Antibacterial, self-healing; tissue-like behavior	Proteins; natural polymers	High bioactivity; controlled release	Complexity in functionalization; high cost	[140]
	Polyacrylamide-Starch Hybrid Hydrogel	Polyacrylamide, Starch, silver nanoparticles (AgNPs)	Eco-friendly; sensitive detection	Synthetic polymer; biopolymer; nanoparticles	High sensitivity; low cost	Limited stability at higher temperatures	[141]

Note: PVA: Polyvinyl alcohol; PEG: Polyethylene glycol; GO: Graphene oxide; MIP: molecularly imprinted polymers; PANI: Polyaniline; PPy: Polypyrrole; PTh: Polythiophene; pCBMA: Poly (carboxybetaine methacrylate); pSBMA: Poly (sulfobetaine methacrylate); AgNPs: silver nanoparticles.

#### 2.2.3. Hybrid Hydrogel Systems

Hybrid hydrogels improve SERS-based food contaminant detection by combining natural polymers’ biorecognition with synthetic polymers’ durability [142]. These materials enhance mechanical strength, chemical stability, and selective analyte interaction. Examples include chitosan–PVA and alginate–PEG systems, which integrate biopolymers’ molecular recognition and biodegradability with synthetic polymers’ durability and tunability. Chitosan–PVA hydrogels have mechanical strengths up to 2.5 MPa with high ion-binding capacity (40–120 mg g^−1^), while alginate–PEG composites remain stable across pH values (2–12), useful for complex food matrices [33,143]. The fabrication of such composite hydrogels can follow either a sequential or a simultaneous mixing process, thus allowing manipulation (e.g., swelling ability, degradation rate, or surface chemistry) of the properties that could influence SERS performance [144,145]. One approach creates interpenetrating polymer networks (IPNs)—entangled polymer networks without cross-linking—formed simultaneously for increased toughness (fracture energies 100–1000 J/m^2^), compressive strength (200–500 kPa), and stress resistance even after multiple freeze–thaw cycles [146,147]. Furthermore, tunable mesh sizes (2–100 nm) can enable selective transport of analytes and excellent delivery to the plasmonic NPs into the hybrid hydrogel, so that once introduced into the hybrid hydrogel, the subtle aspects of the SERS signal amplification can be maximized. Nanocomposite hydrogels incorporate inorganic NPs (zeros, nanosheets, rods) to add functionalities. Common NPs include magnetic iron oxides, silica, carbon nanotubes, quantum dots, and metal oxides. For instance, a hydrogel containing Fe_3_O_4_ iron oxide magnetic NPs at a concentration of 1–10 wt% (5–20 nm) diameter with superparamagnetic behavior (5–50 emu/g of saturation) may permit remote manipulation thus enabling analyte pre-concentration by the application of a focused magnetic force field (0.1–1 T). Particle-independent generic incorporation of NPs can be conducted using a variety of methods, either through transfer and embedment, in situ synthesis, or chemical functionalization [148,149,150]. The spatial distribution (homogeneous, gradient, or clustered) of nanoparticles greatly influences the function of a hydrogel. Specifically, maintaining structural integrity and preventing leaching of the nanoparticles into the food matrix requires strong adhesion properties between the nanoparticles and polymer network, which typically comes with modifications to the surface of the nanoparticles prior to use [151,152]. Multi-responsive hybrid hydrogels, in particular, show potential because they can respond to environmental triggers that may include pH, temperature, or ionic strength. If the hydrogel system contains PNIPAM (like a large number of hydrogels based on the natural biopolymer), they undergo significant alteration of porosity or volume when the parameters are altered. This responsiveness may lead to dual-mode or orthogonal contaminant detection [153,154], especially in an unpredictable food matrix with continually changing parameters such as emulsions, sauces, or fermented products. In short, hybrid hydrogels offer a versatile and robust approach for SERS-based detection of food contaminants. They combine customizable structure with smart responsiveness and compatibility with nanomaterials, thereby offering the potential to analyze food systems that are sensitive, selective, and reproducible.

### 2.3. Structure-Function Relationship in Hydrogels for Enhanced SERS Performance

The efficacy of hydrogels within a food safety context is tied closely to how their structure translates to function. In SERS-based sensing, this means that certain design specifications—specifically, crosslink density, pore structure, and surface charge—can have a profound impact on how well a hydrogel captures analytes, stabilizes plasmonic nanoparticles, and enhances Raman signal intensity. The degree of hydrogel crosslinking (or in other terms, ‘degree of polymerization’, or ‘crosslinking density’) can be considered either as a parameter used to formulate a hydrogel, or as an adjustable feature to consider, which can provide capabilities for enhanced SERS applications. The degree of crosslinking and other structural specifications may be carefully adjusted to modify sensitivity, selectivity, and mechanical stability for specific food matrices or contaminants.

#### 2.3.1. Crosslinking Density

**Figure 3:**
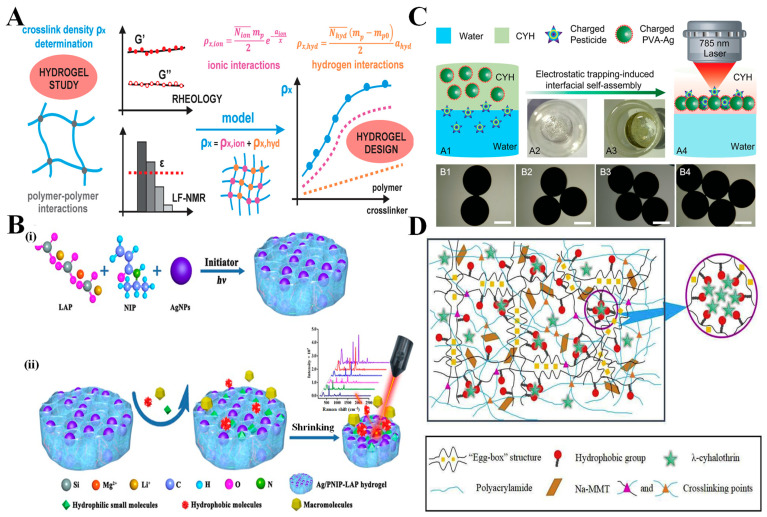
Structural parameters influencing the function and SERS performance of hydrogel systems in food safety detection. (**A**) *Crosslinking density*: Illustration of crosslink density (ρ_x_) as a composite of ionic (ρ_x_,ion) and hydrogen (ρ_x_hyd) interactions, determined through rheology and low-field NMR (LF-NMR). Adjustments in polymer–crosslinker ratios influence network stiffness and permeability, which are critical for analyte diffusion and SERS enhancement. Adapted from [160]; (**B**) *Porosity*: (**i**) Synthesis of a Ag/PNIP-LAP hydrogel through UV initiation; (**ii**) The hydrogel exhibits responsive porosity, expanding to absorb small hydrophilic molecules and contracting against macromolecular interference, providing adaptive sieving and enhanced SERS signal for food contaminants. Adapted from [174]. (**C**) *Surface charge*: Electrostatic trapping at the water-hydrogel interface using positively charged PVA–Ag hydrogel (CYH). A1–A4: Charged pesticides migrate and accumulate at the interface due to electrostatic attraction. B1–B4: Optical images show self-assembly progression, improving hotspot density and Raman signal under laser excitation. Adapted from [41]; (**D**) *Hydrophobicity*: Double-network hydrogel containing polyacrylamide, Na-MMT, and hydrophobic domains structured via an “egg-box” architecture. The matrix selectively traps hydrophobic molecules like λ-cyhalothrin, reducing release rates and increasing SERS sensitivity in lipid-rich food matrices. Adapted from [192].

Crosslinking density can be defined as the number of chemical or physical links made between polymer chains [66,155]. A higher number of crosslinks will create more dense networks with improved mechanical stability (critical for food systems with large imperfections, such as carbonated beverages or acidic products) [156]. Unfortunately, crosslinking on a molecular scale means that increased structural integrity usually implies a decrease in analyte transport. Tightly crosslinked hydrogels will likely have micropores greatly restricted in size, which would slow and possibly prevent contaminants from reaching the plasmonic nanoparticles present and therefore reduce the intensity of any signal acquisition. On the flip side, reduced crosslink density makes networks more permeable and also allows for faster diffusion of the analyte. For example, in the detection of small molecules such as pesticides, crosslinked hydrogels offer a more accurate and rapid detection method for SERS [157]. However, poorly crosslinked hydrogels may jeopardize reproducibility due to soft networks during sample collection [158,159]. Kopač et al. [160] proposed a model detailing how hydrogels behave with variation in crosslink density and pioneered a quantitative dissection of the ionic and hydrogen bonding contributions through rheology and low-field NMR (Figure 3A). Their findings showed that the changes in crosslinker content affect stiffness and mobility of water—both key processes for successfully maintaining SERS stability within complex food matrices. To counteract the suspension of robustness with permeability, designs using gradient crosslinking architecture have drawn from biological tissues like cartilage, whereby the outer layer ensures mechanical safeguard, with the inner core being a permeable element [161,162]. The gradient style design allows the hydrogel to support physical stress while providing effective diffusion [163,164,165]. New fabrication methods have also explored dynamic crosslinking methods that can respond to operating conditions like pH or temperature [166]. Optimizing crosslinking density in hydrogels balances mechanical stability and analyte permeability, directly influencing SERS performance. Techniques like gradient and dynamic crosslinking improve detection efficiency in complex food matrices.

#### 2.3.2. Porosity

Porosity plays an essential role in analyte transport and molecular sieving within hydrogels. Besides pore size, the distribution and interconnectivity of the pores govern whether contaminants can reach the SERS hotspots while maintaining selectivity to exclude larger food matrix components. Novel approaches in coordination chemistry, UV-relaxation chemistry, and stimuli-responsive polymerization allow for the accurate tailoring the pore characteristics of hydrogels [167,168,169]. Pore sizes can be modified depending on the application; mesopores (2–50 nm) are good for isolating small molecule contaminants (i.e., bisphenol A and acrylamide), whereas macropores (>50 nm) absorb samples rapidly and can include bulk diffusion with low selectivity [135,170]. The pore structure will also aid in the enhancement of SERS. Interconnected or multilayered porous structures increase internal light scattering. The scattered light becomes trapped as photons as they continuously bounce off the walls of the structure, further amplifying the local electromagnetic field surrounding the embedded nanoparticles [171,172,173]. This contributes to an intensified Raman signal, making it suitable for hydrogel-nanocomposite systems. A notable example of this was demonstrated by Su et al. [174] utilizing a UV polymerized Ag/PNIP-LAP hydrogel for SERS-based food detection; as seen in Figure 3B, the hydrogel network has an adaptive sieving effect, having the porosity expand when absorbing small hydrophilic molecules through the matrix while also contracting to recognize incoming large food matrix components. Other developments have capitalized on these formations with dual-scale architectures that combine macropores for transport and mesopores for entrapment, resulting in a sensitivity 90% greater when analyzing peanut butter [175]. Other more advanced developments see pH-responsive hydrogels, which will increase porosity in acidic conditions for increased contaminant uptake for food matrices containing vinegar [176,177]. All together, these developments support the necessity and importance of porosity when optimizing SERS performance for complex matrices in food analysis.

#### 2.3.3. Surface Charge

Surface charge is vital in electrostatic interactions with analytes, having a pronounced impact on specificity and capture efficiency. Whichever functional groups are embedded will create electrostatic hotspots by using functional groups like COOH, NH_3_^+^, PO_4_^−^, and SO_3_^−^, and attract complementary charged contaminants [178]. These charged domains act like biological binding pockets, providing the ability to reach detection sensitivities below 1 part per billion for compounds like organophosphate pesticides [179,180]. The action of charge-mediated recognition is more than just simple molecular sieving, since the functional groups introduce a level of selectivity through the introduction of selective electrostatic attraction, providing an increase in specificity and signal clarity. Zhang et al. [41] showed this principle using a positively charged PVA-Ag hydrogel (CYH), attracting and immobilizing negatively charged pesticide molecules at the water-hydrogel interface (Figure 3C). In their system, a positively charged CYH was used to attract and immobilize negatively charged pesticide molecules at the water-hydrogel interface. This electrostatic trapping not only aided in the capture of interacting pesticides but also formed dense analyte-nanoparticle aggregates, which led to a significant increase in SERS signal intensity upon laser irradiation, illustrating how engineered surface charge can alter analyte capture and capture and facilitate a highly sensitive real-time contaminant detection in an aqueous food situation. Compared to biosensors, which often rely on the nearly fragile nature of biological components, charged hydrogels exhibit greater environmental stability, making them suitable for long-term deployment in diverse food matrices such as dairy, seafood, and processed goods. Their reusability and tunability further position them as robust platforms for sustainable and accurate food contaminant monitoring [181]. In conclusion, surface charge in hydrogels enhances analyte specificity and capture efficiency, boosting SERS performance. Functional groups attract contaminants, forming dense analyte-nanoparticle aggregates that improve signal intensity for sensitive, real-time food safety detection.

#### 2.3.4. Hydrophilicity

Hydrophilicity is a crucial variable in the performance of hydrogels, particularly for analytes soluble in water. Water-soluble hydrogel polymers will have an increased water uptake when they have polar functional groups, which allows them to develop aqueous channels for the subsequent diffusion of analytes toward the plasmonic hotspots [182]. Structure-based adjustments, such as crosslinking through transglutaminase, have recently been used in order to increase the hydrophilic capacity of hydrogels, including the water body, swelling rate, and freeze–thaw, is primarily relevant to the food sector, wherein these components were important for a consistent performance [183,184]. Linked to the previously defined hydrophilic domains is the support of molecular recognition through hydrogen bonding and electrostatic interaction; thus, the use of a carboxyl-rich matrix can interact with polar toxins, while amino groups can bind to negatively charged residues [185]. For example, Liu et al. [186]. In highly aqueous food products like milk or fruit juices, hydrophilic hydrogels observed that since hydrophilic hydrogels can trap an aptamer, ultra-sensitive detection (0.51 ppb) of ochratoxin A in wine and milk is possible promote efficient diffusion of polar analytes toward plasmonic hotspots, resulting in stronger and more consistent SERS signals [187]. They also allow a water-rich environment, which stabilizes nanoparticles and diminishes variability of the signal so they can be reliable in real-time applications of food [188,189].

#### 2.3.5. Hydrophobicity

Hydrophobicity has been purposefully introduced into hydrogel matrices for the more selective detection of hydrophobic contaminants, such as lipid-soluble toxins, which are otherwise difficult to detect in aqueous environments, and pesticide residue. The incorporation of hydrophobic groups (e.g., alkyl chains, aromatic rings) results in non-polar domains that adsorb the hydrophobic analytes based on van der Waals forces and hydrophobic interactions [190]. This design was particularly beneficial in analyzing fatty or oil-based food products like dairy, oils, and meat, where hydrophobic contaminants partition into lipid phases [191]. The hydrophobic domains throughout the hydrogel matrix are distributed in a fashion that concentrates the target analyte (e.g., pesticide residues) in close proximity to the plasmonic nanoparticles and increases the SERS signal potency; this occurs because the hydrophobic domains act like molecular sponges that trap the non-polar contaminants at the nanoparticle surface. Wang et al. [192] demonstrated this by incorporating hydrophobic groups in a modified alginate matrix to regulate the rate of pesticide release. The hydrophobic double-network hydrogel, which was reinforced with montmorillonite (MMT) (Figure 3D), created microdomains within the structure that preferentially trapped hydrophobic molecules like λ-cyhalothrin, and therefore showed reduced pesticide leach rates of only 6.68% over 87 h, for total controlled release. Hydrophobicity can be actively modulated dynamically as well. Hydrogels showing a combination of hydrophilic and hydrophobic characteristics also have the potential to stabilize emulsified systems and allow selective interactions. For example, in salad dressings, hydrophobic domains stabilize interactions with lipid phases, while hydrophilic regions maintain structural integrity in aqueous conditions [193]. In contrast, for fatty or oil-rich food products such as meat or dairy, hydrophobic hydrogel domains facilitate the selective capture and concentration of non-polar analytes (e.g., lipid-soluble pesticides), enhancing the SERS signal through localized enrichment [194]. This approach improves SERS signal strength, especially in fatty food products like dairy and oils.

#### 2.3.6. Nanoparticle Distribution

The distribution of nanoparticles within hydrogel matrices has a vital influence on the sensitivity, stability, and reproducibility of SERS-based detection of food contaminants. The best-case scenario for nanoparticle distribution provides uniformly distributed electromagnetic hotspots—local regions of enhanced electric fields caused by plasmonic coupling—to amplify the Raman signal from the analyte molecules [195]. A consistent state of particle dispersion is essential for reliable performance. Overcrowding leads to quenching and variability, whereas excessive spacing between nanoparticles weakens plasmonic coupling, resulting in a more stable but less efficient signal [196]. Therefore, reproducing the proper distribution of nanoparticles within hydrogel network systems is important for reliable and sensitive detection. A number of more advanced fabrication techniques have been developed in order to control the placement of nanoparticles. Radical polymerization, for example, provides the ability to grow successive polymer chains around the pre-percolated nanoparticle environment. This control allows the interparticle distances to be controlled at the nanometer scale (2–5 nm typically), which will maximize the plasmonic coupling potential without promoting excessive aggregation [187]. Alternatively, nanoprecipitation techniques can be used to incorporate nanoparticles into hydrophobic domains and limit nanoparticles from migrating out while keeping a stable semi-disperse distribution [197]. These techniques provide opportunities for transformative applications; hydrogels with well-dispersed silver nanoparticles (AgNPs) have been described for ultra-sensitive detection of contaminants such as melamine in infant formula at concentrations less than 0.01 ppm [198]. In addition, hydrogels with gradient-distributed AgNPs in a hydrogel matrix provided reproducibility of signals at over 95% within batches of contaminants [48].

#### 2.3.7. Network Relaxation

Network relaxation relates to the reconfiguration of the hydrogel’s polymeric network in response to changes in its environment or conditions, often referred to as environmental responsiveness. Hydrogel’s environmental responsiveness (and its dynamic property) is vital, allowing food to rapidly change as the system changes and for the effects of each analyte’s transport response, the concentration of target molecules close to plasmonic hotspots, and the preservation of embedded nanoparticles [199]. This concept of network relaxation and plasticity is particularly beneficial in food systems with a mixture of physiochemical conditions. This process is mostly observed with pH-responsive hydrogels. They show a notable swelling or shrinking when exposed to acidic food will give place to other major pH changes, like fermented milk products, for example. Lactic acid bacteria rapidly lower the pH, inducing swelling or dimensional changes in the hydrogel. This process exposes plasmonic nanoparticles that were previously physically shielded within the hydrogel. This allows for the SERS signal, in this case histamine, to increase and enhance its capture [200]. Thus, it projects a very rapid responsiveness to pH, which could conserve the fluctuation of transient contaminants. Conversely, slow relaxation kinetics could allow for transient contaminants to pass. If the device’s network rigidity was too sturdy, network relaxation would not focus analytes sufficiently for SERS capture. pH-responsive hydrogels allow for certain conditions. If the hydrogel had been exposed to high ionic strength solutions, such as sauces, soups, and brines, the nanoparticles may aggregate in an uncontrolled manner, and the hydrogel with high ionic strength would stabilize the homogenized dispersion of the nanoparticles [201]. Hydrogels having charge density could change pore dimensions and change electrostatic reactions as a function of the ionic conditions of the product to inhibit the clustering of the nanoparticles and thus promote signal reproducibility [202]. Zhang and Silverstein designed and developed very porous zwitterionic hydrogels as an emulsion template with an “anti-polyelectrolyte effect”, where their uptake performance may increase even in NaCl salt levels. This demonstrates their degree of structural responsiveness and stabilization even in high salinity process conditions, making them useful candidates for use in high salinity food matrices [203].

#### 2.3.8. Pore Connectivity

Pore connectivity in hydrogel matrices dictates the transport of analytes, solvents, and nutrients, which affects the efficiency of the detection of contaminants. This feature acts as a highway system, allowing for the rapid diffusion of target molecules towards plasmonic nanoparticles as a continuous transport network. Greater pore connectivity allows for transforming analytes—such as bacterial pathogens, toxins, and residues—to more easily transit the hydrogel network and discover plasmonic hotspots. Poorly connected, and/or isolated pockets of porosity hinder analytes from accessing plasmonic hotspots; the larger the transit distance for an analyte to diffuse, the less sensitivity and increased response time there will be [204,205]. A number of fabrication techniques modify the pore connectivity of hydrogels. One such technique is freeze-drying. In freeze-drying, an ice-porous network is created and then sublimated, leaving behind pathways for analyte movement to occur. Wahid et al. [206] demonstrated that freeze-dried carboxymethyl chitosan hydrogels showed the ability for bacterial diffusion to improve and also increased prevention of bacterial growth against Escherichia coli and Staphylococcus aureus. Electrospinning is another technique that provides webbed nanoscaffolds with open-pore networks that enable analyte movement through molecular diffusion and increased sensitivity to bacterial detection in enzyme-triggered biosensing [207]. The ideal pore connectivity is the balance of analyte diffusion and structural integrity. Hydrogels with ideal pore connectivity have also been used for real-time SERS detection of Salmonella on poultry processing conveyor apparatus to replace traditional laboratory methods [208]. Modifying pore connectivity in hydrogel matrices positively impacts the sensitivity of SERS.

## 3. Advantages of Hydrogel-Integrated SERS Platforms in Food Safety Detection

**Figure 4:**
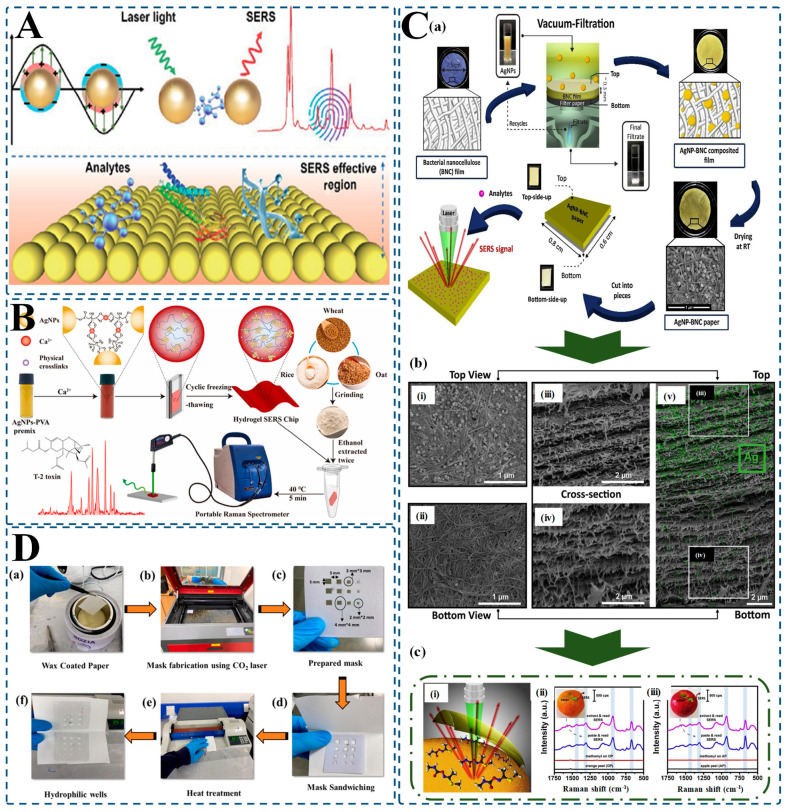
Functional advantages of hydrogel-integrated SERS platforms for enhanced food safety detection. (**A**) Schematic of the SERS effect: laser excitation induces localized surface plasmon resonance between plasmonic nanoparticles, generating electromagnetic “hot spots” that amplify Raman signals from analytes [212]; (**B**) A hydrogel-based SERS chip developed with AgNPs-PVA for T-2 toxin detection: cyclic freeze-thawing enables the formation of a stable matrix, allowing for the sensitive detection of toxins in grain extracts with a portable Raman spectrometer [217]; (**C**) (**a**) Fabrication of flexible AgNP–BNC (bacterial nanocellulose) composite paper via vacuum filtration; integration of AgNPs into BNC film ensures stable, sensitive SERS response; (**b**) (**i**–**v**) SEM images showing top view, bottom view, and cross-sections of AgNP–BNC paper structure, highlighting embedded AgNPs and uniform distribution for high SERS activity; (**c**) (**i**) Schematic of in situ detection mechanism; (**ii**,**iii**) Raman spectra showing sensitive detection of methomyl pesticide on real fruit surfaces. [218]; (**D**) Flexible microfluidic paper-based SERS sensor fabrication: (**a**–**f**) Step-by-step process from wax-coated paper to heat-treated hydrophilic wells incorporating Ag-decorated graphene oxide for ultra-sensitive analyte capture and signal enhancement. Final spectra show successful detection of contaminants with high reproducibility and long-term stability [221].

SERS stands as one of analytical chemistry’s most powerful detection methodologies, offering remarkable sensitivity that approaches single-molecule detection thresholds. At its core, SERS works through an elegant physical phenomenon: when target molecules interact with plasmonic nanostructures embedded in the sensing substrate, their Raman signals undergo extraordinary amplification [209,210,211]. The “hot spots”, areas of amplified electromagnetic field strength in between nanoparticles, can enhance the intensity of the Raman signal by 10^6^–10^10^, allowing for detection even at ultra-low analyte concentrations (Figure 4A). Although the potential of SERS is enormous, traditional SERS substrates continue to suffer from challenges of unpredictable nanoparticle aggregation and poor reproducibility, and repeatability between samples or different environments [35,212,213,214].

The integration of hydrogels with plasmonic nanoparticles represents a transformative approach that addresses these fundamental limitations through multiple synergistic mechanisms. Hydrogels serve as three-dimensional architectural frameworks that immobilize nanoparticles in precisely controlled spatial arrangements, preventing the random aggregation that typically undermines traditional SERS substrates. This stabilization ensures nanoparticles maintain optimal interparticle distances for maximum electromagnetic field enhancement, dramatically improving signal reproducibility—a critical parameter for reliable quantitative analysis. Traditional SERS substrates often yield relative standard deviations (RSDs) that are greater than 20%, and the hydrogel-integrated designs have achieved RSDs less than 7%, providing a better capacity to determine concentrations [15,215,216].

Beyond stabilization, the precisely engineered porosity of hydrogels creates an intelligent molecular filtration system that selectively permits target analytes to diffuse toward plasmonic hot spots while blocking larger interfering components commonly found in complex food matrices. Chen and colleagues demonstrated this capability with a polyvinyl alcohol hydrogel-based SERS chip specifically designed to admit small toxin molecules while excluding larger food components (Figure 4B). This intelligent filtration enabled detection of T-2 toxin at concentrations as low as 0.41 ppb with exceptional reproducibility (RSD: 2.8–6.7%), even in complex food matrices where conventional methods would be overwhelmed by interference [217]. The inherent flexibility and conformational adaptability of hydrogels enable direct, non-destructive analysis of food products in their natural state. Parnsubsakul and colleagues leveraged this property by developing flexible SERS substrates using bacterial nanocellulose embedded with silver nanoparticles (Figure 4C). These substrates could be directly applied to fruit peels for in situ detection of methomyl pesticide residues without damaging the fruit or requiring complex extraction procedures, demonstrating robust SERS activity while maintaining mechanical integrity during application and removal [218].

Hydrogels also offer a versatile chemical platform for introducing specific molecular recognition elements that enhance target binding affinity and detection specificity. Ouyang and colleagues exploited this capability by encapsulating β-cyclodextrin-modified silver nanoparticles within a polyvinyl alcohol hydrogel matrix. The β-CD molecules served dual functions as reducing agents during nanoparticle synthesis and as molecular recognition elements that improved binding affinity toward sulfonamide antibiotics. This multifunctional approach achieved detection limits as low as 10 ng/mL in complex samples—a level of sensitivity rarely achieved with conventional SERS substrates lacking specific recognition elements [219]. The stimulus-responsive nature of certain hydrogels introduces an additional dimension of functionality. These “smart” hydrogels can dynamically respond to environmental triggers such as pH, temperature, or ionic strength—conditions that often vary significantly across different food matrices. By harnessing these responsive properties, hydrogel-SERS platforms can adapt to specific detection environments, optimizing their performance for particular food systems [220].

Furthermore, the versatility of hydrogels facilitates their integration with complementary technologies, creating multifunctional detection platforms. Naqvi and colleagues developed a flexible microfluidic SERS sensor combining hydrophilic wells on paper with silver nanoparticle-decorated graphene oxide (Figure 4D). The hydrophilic wells enabled precise analyte trapping, achieving remarkable sensitivity—detecting thiram at concentrations as low as 0.36 × 10^−16^ M with an enhancement factor of 5.2 × 10^12^. The sensor showed high reproducibility (RSD < 5%) and long-term stability, with intensity deviation < 4% over two months [221]. Similarly, Kim et al. [222] engineered bimetallic Au–Ag nanowires with an ultrathin hydrogel skin that effectively excluded large food components while permitting target molecules to reach plasmonic hot spots, achieving detection limits as low as 10 ppb for tricyclazole pesticide even in whole milk.

Perhaps most significantly, hydrogel-based SERS platforms address the critical gap between laboratory capabilities and field deployment. Their mechanical stability, ease of handling, and compatibility with portable Raman spectrometers make them ideally suited for on-site analysis in food production environments. The long-term stability demonstrated by many hydrogel-SERS systems—with some maintaining consistent performance for months under ambient conditions—eliminates the need for specialized storage procedures, while their ability to function without elaborate sample preparation reduces the technical expertise required for operation. These combined advantages position hydrogel-integrated SERS platforms as transformative tools for food safety monitoring—bridging the gap between analytical sensitivity and practical applicability that has long challenged the implementation of advanced detection technologies in real-world food systems. As research continues to refine these materials, we can anticipate even more sophisticated hydrogel-SERS platforms that further enhance the detection of contaminants in our increasingly complex global food supply.

## 4. Applications in Food Safety Detection

Table 2 summarizes recent studies that utilize hydrogel-based SERS systems for detecting contaminants in foods. The following sections explore material designs and fabrication strategies tailored to overcome the specific challenges faced in food safety monitoring.

### 4.1. Food Adulteration and Fraud Detection

Food adulteration—encompassing the contamination, substitution, or dilution of products with undeclared and potentially harmful substances—poses significant risks to both public health and market integrity [223,224]. Common examples include the addition of melamine to inflate protein content artificially or the use of toxic dyes like Sudan IV in spices to enhance their appearance. Such practices can have serious health repercussions, ranging from organ damage and allergic reactions to an increased risk of cancer. In addition, food fraud erodes consumer trust and imposes considerable economic burdens on industries and regulatory agencies due to product recalls and compliance costs [225]. As adulteration methods grow more sophisticated, many conventional analytical techniques are no longer sufficient, creating an urgent need for advanced, rapid, and dependable detection platforms that perform effectively in complex food matrices. In this context, SERS integrated with hydrogel-based substrates offers an effective solution for detecting food adulteration.

**Figure 5:**
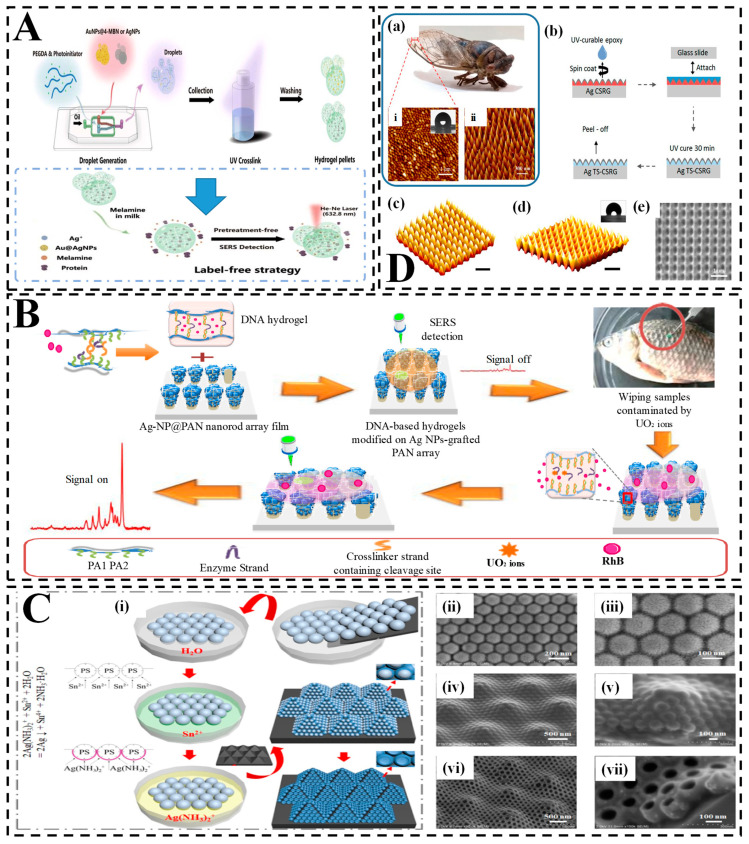
Hydrogel-integrated SERS platforms for detecting food adulteration and fraud. (**A**) Schematic of a label-free SERS detection system using Au@AgNP-MBN-loaded PEGDA hydrogel micropellets. Droplet generation followed by UV crosslinking creates porous pellets enabling sensitive detection of melamine in milk without pretreatment [226]; (**B**) DNAzyme-functionalized hydrogel SERS chip for detecting uranyl ions in fish. The smart hydrogel capsule undergoes conformational change upon DNA–ion interaction, triggering a switchable SERS signal. The process involves selective analyte capture, signal on/off switching, and real sample testing on fish (highlighted area) [227]; (**C**) Fabrication of a light-trapping hydrogel SERS substrate inspired by compound eyes; (**i**) Self-assembly of Ag(NH_3_)_2_⁺ nanostructures into periodic arrays via nanosphere lithography and ion-assisted deposition; (**ii**–**vii**) SEM images at different magnifications show uniform nanostructures critical for enhancing SERS signal via light confinement and plasmonic coupling [228]; (**D**) Cicada-wing-inspired nanostructured substrate for melamine detection; (**a**) Image of a cicada and its wing nanostructures (**i**: macroscopic; **ii**: AFM of surface); (**b**) Fabrication of Ag-coated cicada-structured replica glass (Ag TS-CSRG) using UV-curable epoxy; (**c**–**e**) Schematic renderings of surface topographies used to replicate and optimize light trapping and SERS enhancement [98].

One noteworthy example is the work of Sun et al. [226], who developed SERS-active hydrogel micropellets through ultraviolet polymerization, embedding citrate-reduced gold nanoparticles within a three-dimensional porous matrix (Figure 5A). The microporous hydrogel structure facilitated the formation of uniform plasmonic hot spots while excluding larger interfering molecules. This design enabled sensitive detection of melamine in milk, achieving a detection limit of 10 nM, alongside excellent signal stability and reproducibility—qualities well-suited for routine food safety screening.

Building on this concept, He et al. [227] created a hydrogel system functionalized with DNAzyme and integrated it into silver nanoparticle-coated polyacrylonitrile (PAN) nanorod arrays for detecting uranyl ions in fish (Figure 5B). Acting as a smart sensing capsule, the hydrogel selectively captured and released Raman reporters in response to DNA targets tied to mislabeled seafood species. This approach achieved picomolar-level sensitivity and provided a robust platform for seafood traceability and authentication.

Meanwhile, Jin et al. [228] introduced an innovative light-trapping hydrogel-SERS substrate inspired by the compound eye structures of insects (Figure 5C). They fabricated the hydrogel matrix using nanosphere lithography and anisotropic etching to form silicon micropyramidal arrays, then assembled silver nanobowls onto these micropyramids via liquid–solid interface self-assembly. The resulting hierarchical substrate enabled ultrasensitive detection of four synthetic dyes—crystal violet, malachite green, methylene blue, and rhodamine 6G—with excellent specificity and selectivity, even in complex food matrices.

Additionally, Nair et al. [98] developed a hydrogel-SERS biosensor inspired by cicada wing nanostructures to detect melamine adulteration in milk (Figure 5D). Fabricated using holographic exposure and template-assisted stripping, this nanostructured hydrogel featured self-cleaning and anti-biofouling properties. Its hierarchical surface architecture enhanced plasmonic coupling and delivered robust signal amplification. With a detection limit of 1 ppm for melamine, this system offers a rapid, cost-effective alternative to conventional chromatography-based methods.

Together, these studies highlight the power of hydrogel-SERS platforms in food adulteration and fraud detection. By leveraging biomimetic surface structures, intelligent molecular interactions, and hierarchical nanoarchitectures, researchers have achieved unparalleled levels of sensitivity and specificity. Ultimately, the fusion of hydrogels with SERS paves the way for real-time, on-site food safety monitoring, marking a significant step toward next-generation rapid authentication technologies.

**Table 2:** Hydrogel systems coupled with SERS for food safety applications.

Analyte	Hydrogel-Based Substrate	Type of Material	Fabrication Method	Plasmonic Metal	LOD	Relative Standard Deviation (RSD)	Real Sample	Reference
Crystal violet	Cicada wing	Natural	Magnetron sputtering	Cu@Ag core–shell NP	10^−10^ M	<15%	-	[229]
Melamine and thiamethoxan	Chitosan film	Natural	Direct Incorporation	AuNPs	1.5 mg.kg^−1^ (melamine) 0.001 mg.kg^−1^ (thiamethoxan)	5.66%	Milk and apple surfaces, respectively	[230]
6-benzylaminopurine auxin and 4-amino-5,6-dimethylthieno (2,3-d) pyrimidin-2(1H)-one hydrochloride additive	Cicada wing	Natural	Magnetron sputtering	Au@Ag nanodome-cones array (Au@Ag NDCA)	38.8 μg/L (6-benzylaminopurine auxin) 18.0 μg/L (4-amino-5,6-dimethylthieno (2,3-d) pyrimidin-2(1H)-one hydrochloride additive	1.5–6.5% and 3.5–7.9% respectively	Sprout and beverage, respectively	[99]
Glucose monitoring	3D Hydrogel-SERS chip	Natural	Encapsulated probe molecules in nanoscale gaps	Gold nanoflower-like nanotags	0.25 mM (glucose)	2.58%	Sweet	[231]
Melamine	Ag nanoparticles in agar gel hydrogel	Hybrid	In situ reduction method	AgNPs	10^−15^ M	7.58%	Camel milk powder	[198]
Rhodamine and crystal violet	Graphene oxide hydrogel	Synthetic	Hydrothermal self-assembled of GO nanosheets into GO hydrogel	AuNPs	10^−8^ M		-	[232]
Phenolic residues	Polyacrylamide-Starch Hybrid Hydrogel	Hybrid	Silver nanoparticles encapsulated in hybrid hydrogel	AgNPs	1 × 10^−8^ M	-	Water	[141]
Streptomycin	DNAzyme crosslinked DNA hydrogel	Natural	Controlled evaporation self-assembly of AuNRs + Thermal annealing DNA hybridization	Au nanorods (AuNRs)	4.85 × 10^−3^ nM	0.16–2.55% and 2.11–3.60% respectively	Honey, milk	[233]
Sudan Red (SR) III	Poly(vinyl alcohol) (PVA) hydrogel	Synthetic	Conformal hydrogel SERS substrate	AgNPs	1.6 ng/4 cm^2^	-	Fruit peel (kumquat)	[234]
Cyanine5 (Cy5) acid dye	Metal film over polyimide nanopillars (MFPNs) with hydrogel encapsulation	Synthetic	Maskless plasma etching and hydrogel coating	Silver (200 nm) + Gold (5 nm)	100 pM	10.0%	Aqueous samples	[235]
Rhodamine 6G, Thiram, 2-naphthalenethiol	Bacterial nanocellulose (BNC) with in situ AgNPs	Natural	Silver mirror reaction and volume shrinkage treatment	AgNPs	1.6 × 10^−8^ M (R6G), 3.8 × 10^−9^ M (Thiram)	10.8%	Organic pollutants in solution	[236]
*E. coli*	Chitosan hydrogel	Natural	Hydrogel crosslinked with glutaraldehyde combined with aptamer-modified gold nanostars	AuNPs	3.46 CFU/mL	14.0–15.2% and 13.2–22.2% respectively	Milk, juice	[237]
Kanamycin (antibiotic)	DNA hydrogel	Synthetic	Ligation-rolling circle amplification	Au@Au core–shell NP	2.3 fM	1.6–7.9%	Milk, honey	[238]

Note: LOD: Limit of detection; RSD: relative standard deviation; NP: nanoparticle; AuNPs: gold nanoparticles; AgNPs: silver nanoparticles; AuNRs: gold nanorods; Au@Ag NDCA: gold-silver nanodome-cones array; GO: graphene oxide; BNC: bacterial nanocellulose; PVA: Poly(vinyl alcohol); MFPNs: metal film over polyimide nanopillars; Cy5: Cyanine 5; R6G: rhodamine 6G.

### 4.2. Pesticide Detection

**Figure 6:**
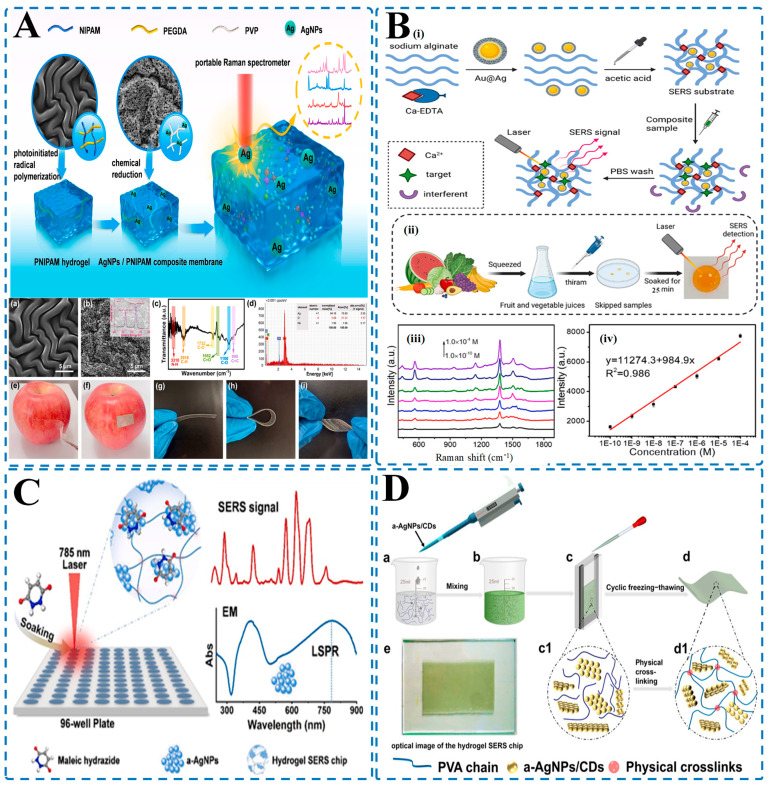
Hydrogel-integrated SERS platforms for rapid and sensitive pesticide residue detection in food samples. (**A**) SERS platform based on a PNIPAM/PEGDA/PVP hydrogel composite embedded with AgNPs ((**a**) Surface morphology of PNIPAM hydrogel; (**b**) SEM image of AgNP-loaded PNIPAM composite; (**c**) Raman spectra showing SERS activity of the composite hydrogel; (**d**) Elemental mapping confirming Ag presence; (**e**,**f**) Visual demonstration of hydrogel patch on apple for direct pesticide detection; (**g**–**i**) Flexibility of hydrogel membrane enabling surface adaptability [246]); (**B**) Sodium alginate hydrogel embedded with Au@Ag nanoparticles for thiram detection in juice samples ((**i**) Working principle of target-specific ion crosslinking and selective SERS sensing using Ca²⁺ and PBS washing; (**ii**) Workflow showing sample preparation from squeezed fruit juices and direct detection; (**iii**) Raman spectra for thiram at different concentrations (10^−10^–10^−4^ M); (**iv**) Calibration curve demonstrating excellent linearity and sensitivity [143]); (**C**) Hydrogel-based SERS platform for detecting maleic hydrazide using a-AgNPs in a 96-well format. Soaking and 785 nm laser irradiation generate strong electromagnetic (EM) enhancement and localized surface plasmon resonance (LSPR), enabling high SERS signal readout [49]; (**D**) Fabrication of a hydrogel SERS chip integrating a-AgNPs/CDs (carbon dots)((**a**–**d**) Steps including nanoparticle mixing, casting, freezing–thawing, and final chip formation; (**c1**,**d1**) Schematic of internal physical crosslinking network; (**e**) Optical image of completed hydrogel chip for real sample application [48]).

Pesticides are essential components of modern agriculture, protecting crops from pests, weeds, and diseases, thereby ensuring global food security [239,240]. However, residual pesticides on agricultural products present significant health hazards to consumers, including cancer, reproductive toxicity, developmental disorders, and endocrine disruption [241,242]. To protect public health and preserve ecological balance, international regulatory agencies have established maximum residue limits (MRLs) for pesticides in food products. Despite these regulations, detecting pesticide residues remains challenging due to their extremely low concentrations and the complex nature of food matrices [243,244,245]. Several studies have utilized hydrogel-based SERS platforms as particularly promising solutions, offering rapid, sensitive, and reproducible pesticide detection without extensive sample preprocessing:

Zhang et al. [246] created a flexible SERS substrate by incorporating polyvinylpyrrolidone-stabilized silver nanoparticles (PVP-AgNPs) within a poly(N-isopropylacrylamide) (PNIPAM) hydrogel matrix (Figure 6A). This innovative system successfully detected methomyl pesticide on apple surfaces at concentrations as low as 10^−9^ M. The platform demonstrated exceptional signal uniformity with a relative standard deviation below 10% while maintaining excellent mechanical flexibility for direct surface sampling.

Qi et al. [143] employed a complementary approach by embedding gold-silver core–shell nanoparticles (Au@Ag NPs) within a sodium alginate hydrogel framework (Figure 6B). The three-dimensional porous structure of the hydrogel facilitated uniform nanoparticle distribution, resulting in highly consistent SERS hotspots and enhanced mechanical adaptability. This system achieved direct, label-free detection of thiram residues in fruit juice at an impressive detection limit of 1 × 10^−10^ M without requiring complex sample preparation steps.

Fu et al. [49] enhanced hydrogel-based SERS sensitivity by integrating aggregated silver nanoparticles (a-AgNPs) into a polymeric hydrogel network specifically designed for detecting maleic hydrazide (MH) in tea and potato samples (Figure 6C). The hydrogel matrix effectively minimized interference from complex food components, achieving a practical detection limit of 50 ppb with recovery rates ranging from 92.80% to 105.6%. Notably, this system completed analysis within just 10 min, demonstrating its potential for rapid field testing.

Chen et al. [48] developed an advanced hydrogel-based SERS chip by incorporating single-layer carbon dot-capped silver nanoparticle aggregates (a-AgNPs/CDs) into a polyvinyl alcohol (PVA) hydrogel matrix (Figure 6D). This sophisticated platform demonstrated remarkable sensitivity, detecting thiram and 2-(4-thiazolyl) benzimidazole at extremely low concentration limits—1 ppb in liquid samples and 1 ng/cm^2^ on fruit surfaces—making it suitable for practical food safety monitoring.

These research advances collectively demonstrate the effectiveness of hydrogel-based SERS platforms for identifying trace pesticide residues in agricultural products. By providing rapid, sensitive, and straightforward detection methods, these innovative systems represent a promising alternative to conventional analytical techniques. Their continued development and implementation will support safer food production practices and facilitate compliance with increasingly stringent regulatory standards worldwide.

### 4.3. Detection of Food Toxins and Illegal Additives

The detection of toxic compounds and illegal additives in food is critical for consumer safety. These harmful substances present significant health hazards including carcinogenic, mutagenic, and neurotoxic effects [247,248]. Unauthorized dyes like Sudan IV and compounds like melamine enhance appearance or falsely elevate nutritional measurements, often with devastating health consequences.

**Figure 7:**
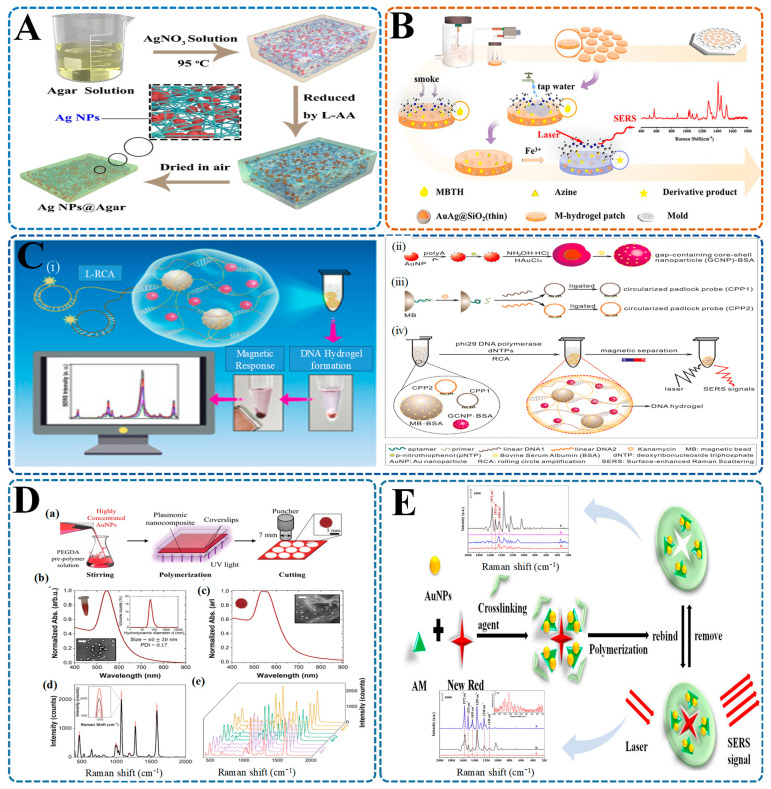
Hydrogel-integrated SERS platforms for detecting food toxins and illegal additives. (**A**) AgNPs@Agar hydrogel biosensor for melamine and penicillin G detection in camel milk powder. Silver nanoparticles are formed via in situ reduction with L-ascorbic acid in an agar matrix, creating a flexible SERS-active hydrogel membrane [198]; (**B**) Agarose hydrogel-SERS patch for formaldehyde (FA) detection using AuAg@SiO_2_ nanoparticles. The hydrogel is functionalized with MBTH to form a derivative product upon reacting with FA, producing a strong Raman signal at 1273 cm^−1^ under laser excitation [36]; (**C**) DNA hydrogel-based SERS platform for kanamycin detection using ligase-rolling circle amplification (L-RCA) ((**i**) L-RCA amplification forms a DNA hydrogel with magnetic responsiveness for SERS sensing; (**ii**) Synthesis of aptamer-functionalized gold nanoparticles (polyA-AuNPs) and preparation of gold core–shell SERS tags (GCNPs) with encapsulated Raman reporters; (**iii**) Hybridization of circularized padlock probes (CPP1 and CPP2) and ligation to form a double-targeted detection structure for kanamycin; (**iv**) Phi29 DNA polymerase-driven amplification and magnetic bead separation produce a DNA hydrogel embedded with GCNPs for enhanced SERS detection [238]). (**D**) PEGDA-AuNP nanocomposite hydrogel chip for detecting sulfur dioxide (SO_2_) in wine ((**a**) Fabrication of the SERS chip using UV curing and punching to produce 7 mm discs; (**b**,**c**) UV–Vis spectra showing localized surface plasmon resonance of AuNP-loaded hydrogels; (**d**) SERS spectra under different SO_2_ concentrations; (**e**) 3D Raman intensity plots confirming detection sensitivity [257]). (**E**) Molecularly imprinted hydrogel (MIH) for New Red azo dye detection. The hydrogel is synthesized with New Red as a template, forming specific binding cavities. After template removal, rebinding of the dye enables selective SERS signal generation with high recovery rates in beverage samples [259].

Melamine, a nitrogen-abundant chemical illicitly added to food to boost apparent protein content, poses kidney damage risks [249,250]. Sodium penicillin G misuse in livestock contributes to antimicrobial resistance. Recognizing these hazards, Chang et al. [198] engineered a three-dimensional hydrogel biosensor with silver nanoparticles in an agar matrix (AgNPs@Agar) for detecting both contaminants in camel milk powder (Figure 7A). This hydrogel, fabricated through in situ reduction, achieved sensitivity of 10^−15^ M for targeted molecules while maintaining performance over 49 days. This platform shows promise for field-deployable detection of unauthorized additives in dairy products.

Formaldehyde (FA), a carcinogen found in water from industrial and agricultural sources, threatens food safety. Cao et al. [36] developed an agarose hydrogel-SERS patch with AuAg@SiO_2_ nanoparticles for FA detection (Figure 7B). This biomimetic system, functionalized with 3-methyl-2-benzothiazolinone hydrazine (MBTH), selectively captures formaldehyde, generating an amplified Raman signal at 1273 cm^−1^. The platform achieves a detection limit of 2.92 × 10^−5^ mg/m^3^, providing reliable monitoring for food safety applications.

Antibiotic compounds, particularly kanamycin, are extensively utilized in contemporary food production systems to combat bacterial infections in livestock, yet their unauthorized or excessive presence in consumable products creates serious public health concerns, most notably the acceleration of antimicrobial resistance development [251,252,253,254]. Chen et al. [238] developed a DNA hydrogel-SERS aptasensor for detecting kanamycin residues. Using ligase-rolling circle amplification (L-RCA), they created a DNA hydrogel network capturing Au@Au core–shell nanoparticles (GCNPs), enhancing Raman signals for kanamycin detection at concentrations as low as 2.3 fM (Figure 7C). This sensitive system is ideal for monitoring antibiotic contamination.

Sulfur dioxide (SO_2_) and sulfites serve as preservatives in winemaking but raise health concerns requiring regulatory oversight and accurate quantification methods [255,256]. Yilmaz et al. [257] developed a hydrogel-based SERS sensor with poly (ethylene glycol) diacrylate (PEGDA) nanocomposites and gold nanoparticles (Figure 7D). By adjusting wine pH to 10, they enabled direct SO_2_ detection down to 0.4 ppm, providing cost-effective and sensitive sulfite monitoring in wine production.

New Red is a synthetic azo dye commonly used as a food colorant to enhance the visual appeal of processed foods and beverages [258]. However, excessive consumption of this additive has been associated with potential health risks, including allergic reactions and possible carcinogenicity, making its monitoring essential for food safety. Neng et al. [259] created a molecularly imprinted hydrogel (MIH) integrated with SERS to detect New Red in beverages (Figure 7E). This hydrogel, polymerized with New Red templates, creates binding cavities complementary to the dye structure, achieving a detection limit of 1.64 × 10^−7^ M with recovery rates of 86.3–100.6% in contaminated samples.

These examples highlight the versatility of hydrogel-based SERS systems for detecting harmful toxins and unauthorized additives in food. Their rapid, sensitive, and selective response mechanisms provide powerful tools for food safety monitoring, offering advantages over traditional analytical methods. These technologies promise to strengthen food safety infrastructure through enhanced detection capabilities and broader testing accessibility.

## 5. Challenges and Future Prospects

Hydrogels for SERS-based food safety detection represent a transformative advance in analytical technologies, merging diverse material design principles with sophisticated engineering strategies. These platforms transcend conventional boundaries by integrating synthetic and naturally inspired architectures to create sensing environments that stabilize nanoparticle interactions, optimize electromagnetic signal enhancement, and selectively enrich target analytes. Whether employing synthetic polymers, hybrid composites, or biomimetic matrices, these multifunctional systems demonstrate remarkable adaptability for addressing complex food safety challenges across diverse matrices and contaminant classes [99]. Despite these impressive technological achievements, several fundamental challenges remain before hydrogel-SERS systems can successfully transition from laboratory concepts to widespread commercial applications.

(i) Signal reproducibility presents a particularly persistent challenge when moving from controlled research environments to real-world food matrices with heterogeneous compositions of proteins, lipids, carbohydrates, and various additives. This complex chemical landscape can dramatically alter detection reliability, necessitating comprehensive validation under authentic food processing conditions rather than simplified model systems [227]. Future developments should focus on standardizing testing protocols that reflect real-world food matrices and establishing benchmark standards for signal consistency across different sample conditions.
(ii) Manufacturing scalability represents another significant hurdle limiting commercial viability. Laboratory-scale fabrication typically produces small batches with variations in nanoparticle distribution patterns, pore network connectivity, and crosslinking density gradients, leading to inconsistent SERS enhancement factors and compromised reliability across production batches [195]. Addressing this requires developing continuous manufacturing protocols that automate hydrogel synthesis while maintaining precise control over physicochemical properties. Advanced quality control innovations—including in-line spectroscopic monitoring and real-time assessment—could track critical attributes such as viscosity profiles, particle size distributions, and plasmonic activity during fabrication. Furthermore, establishing standardized characterization frameworks with defined acceptance criteria for enhancement factors, signal reproducibility, and environmental stability would provide benchmarks for both batch consistency and cross-manufacturer comparison.
(iii) Long-term stability of plasmonic nanoparticles within hydrogel matrices presents additional barriers. Environmental variables in food systems—including temperature fluctuations, pH shifts, and oxidizing agents—can degrade nanoparticle performance, particularly with easily oxidized silver nanoparticles. Future hydrogel designs could incorporate more sophisticated nanoparticle architectures such as core–shell configurations, protective polymer coatings, or noble metal alloy structures that balance optimal plasmonic enhancement with improved environmental durability. Additionally, integrating stabilizing matrix additives—including antioxidants or metal-chelating agents—could create protective microenvironments around embedded nanoparticles, extending their functional lifespan. Furthermore, strategically designed multilayered hydrogel architectures with robust outer protective layers could shield SERS-active internal regions while permitting selective analyte permeation.
(iv) Translation to accessible detection systems represents another crucial direction for advancement. While SERS analysis traditionally relies on sophisticated, laboratory-based Raman spectrometers, recent developments in miniaturized, smartphone-compatible Raman devices offer promising alternatives for field deployment. Combined with optimized hydrogel substrates, these could support rapid, on-site testing with minimal expertise requirements. Moreover, automated data interpretation algorithms incorporating machine learning approaches could further streamline analysis workflows, enabling real-time decision making at critical control points throughout food production and distribution networks.
(v) Molecular selectivity enhancement remains a priority challenge. Current platforms rely primarily on size exclusion and non-specific charge-based interactions, which may prove insufficient for distinguishing structurally similar analytes in multi-contaminant scenarios. Next-generation designs could incorporate multiple biomimetic recognition elements arranged within spatially segregated detection zones, enabling simultaneous, multiplexed detection of various contaminant classes. Additionally, the integration of molecular recognition elements such as aptamers, antibodies, or molecularly imprinted polymers could significantly improve specificity for target analytes even in complex food matrices.
(vi) Regulatory considerations introduce additional complexity for commercial adoption pathways. Hydrogels incorporating nanomaterials must comply with evolving global food safety regulations, which necessitate thorough evaluation of potential nanoparticle migration, toxicological profiles, and environmental impacts throughout the product lifecycle. Ensuring compliance with international regulatory standards, conducting comprehensive biocompatibility assessments, and examining potential ecological consequences all represent necessary steps toward broader acceptance of these advanced sensing technologies within regulatory frameworks. Key regulatory agencies such as the U.S. Food and Drug Administration (FDA) [260], the European Food Safety Authority (EFSA) [261], and the China National Center for Food Safety Risk Assessment (CFSA) [262] have begun to address the safety of nanomaterials in food applications. However, comprehensive and harmonized international standards specific to hydrogel-nanomaterial hybrids for food detection are still evolving. Development of standardized testing protocols specifically designed for nanomaterial-containing hydrogels would greatly facilitate regulatory approval processes.
(vii) Advanced manufacturing integration shows particular promise for future development. The convergence of hydrogel chemistry with advanced manufacturing techniques—including 3D and 4D printing—enables unprecedented control over nanoparticle organization and hydrogel properties. Programmable hydrogels capable of visible structural or colorimetric changes upon specific analyte detection could provide intuitive visual indicators. Integrating wireless communication and Internet of Things (IoT) capabilities may enable continuous monitoring throughout global supply chains. Multimodal sensing platforms that synergistically combine SERS with complementary techniques like fluorescence spectroscopy or electrochemical methods could enhance detection accuracy through orthogonal verification and reduce false positive or false negative results.

Realizing the full potential of hydrogel-SERS platforms requires coordinated collaboration among academic researchers exploring fundamental properties, industry stakeholders focused on manufacturing, and regulatory bodies establishing appropriate frameworks. By addressing the interconnected challenges of reproducibility, scalability, selectivity, and stability, these advanced materials can evolve into robust sensing solutions for worldwide food safety monitoring, enhancing quality control and consumer protection throughout the global food supply chain.

## 6. Conclusions

This review highlights the significant potential of hydrogel-integrated SERS platforms for food safety detection. Hydrogels, with their unique properties of high-water content, three-dimensional networks, and selective permeability, provide an ideal environment for stabilizing plasmonic nanoparticles and enhancing their signal amplification. Whether derived from natural polymers such as chitosan, alginate, and cellulose, or engineered as synthetic and hybrid composites, hydrogel-based SERS platforms have demonstrated success in detecting a wide range of food contaminants, including pesticides, toxins, and adulterants, even in complex food matrices. Despite these promising advancements, challenges persist, particularly in ensuring reproducibility, long-term stability, and scalable fabrication methods under real-world conditions. The need for improved selectivity in multi-contaminant detection remains crucial, and integrating these systems with portable, user-friendly technologies for on-site analysis is essential. Furthermore, adherence to regulatory standards is vital for the widespread commercial deployment of these technologies. Future research should focus on optimizing the sensitivity, uniformity, and reproducibility of hydrogel-SERS systems while developing versatile substrates for analyte separation and pre-concentration. Material design innovations, such as 3D/4D printing, hold the potential to further enhance these platforms’ performance. As such, hydrogel-based SERS technologies offer a promising path toward rapid, reliable, and accessible food safety monitoring, safeguarding both consumer health and food quality across the global food supply chain. Taken together, hydrogel-SERS platforms represent a transformative advancement in food safety monitoring, offering real-time, sensitive, and portable detection solutions with significant potential for regulatory and industrial integration.
